# Rabbit microbiota across the whole body revealed by 16S rRNA gene amplicon sequencing

**DOI:** 10.1186/s12866-021-02377-x

**Published:** 2021-11-10

**Authors:** Xiaofen Hu, Fei Wang, Shanshan Yang, Xu Yuan, Tingyu Yang, Yunxiao Zhou, Yong Li

**Affiliations:** College of Animal Science and Technology, Jiangxi Agricultural University, Nanchang, 330045 Jiangxi China

**Keywords:** Rabbit, microbial community, whole body sites, 16S rRNA gene

## Abstract

**Background:**

Rabbit can produce meat, fur and leather, and serves as an important biomedical animal model. Understanding the microbial community of rabbits helps to raise rabbits healthily and better support their application as animal models.

**Results:**

In this study, we selected 4 healthy Belgium gray rabbits to collect the microbial samples from 12 body sites, including skin, lung, uterus, mouth, stomach, duodenum, ileum, jejunum, colon, cecum, cecal appendix and rectum. The microbiota across rabbit whole body was investigated via 16S rRNA gene amplicon sequencing. After quality control, 46 samples were retained, and 3,148 qualified ASVs were obtained, representing 23 phyla and 264 genera. Based on the weighted UniFrac distances, these samples were divided into the large intestine (Lin), stomach and small intestine (SSin), uterus (Uter), and skin, mouth and lung (SML) groups. The diversity of Lin microbiota was the highest, followed by those of the SSin, Uter and SML groups. In the whole body, *Firmicutes* (62.37%), *Proteobacteria* (13.44%) and *Bacteroidota* (11.84%) were the most predominant phyla. The relative abundance of *Firmicutes* in the intestinal tract was significantly higher than that in the non-intestinal site, while *Proteobacteria* was significantly higher in the non-intestinal site. Among the 264 genera, 35 were the core microbiota distributed in all body sites. Sixty-one genera were specific in the SML group, while 13, 8 and 1 were specifically found in the Lin, SSin and Uter groups, respectively. The Lin group had the most difference with other groups, there were average 72 differential genera between the Lin and other groups. The functional prediction analysis showed that microbial function within each group was similar, but there was a big difference between the intestinal tracts and the non-intestinal group. Notably, the function of microorganism in uterus and mouth were the most different from those in the gastrointestinal sites; rabbit’s coprophagy of consuming soft feces possibly resulted in little differences of microbial function between stomach and large intestinal sites.

**Conclusion:**

Our findings improve the knowledge about rabbit microbial communities throughout whole body and give insights into the relationship of microbial communities among different body sites in health rabbits.

**Supplementary Information:**

The online version contains supplementary material available at 10.1186/s12866-021-02377-x.

## Introduction

Rabbits are small mammals belonging to the family of Leporidae of the order Lagomorpha, have a nearly worldwide distribution [1]. The domestic rabbit is one of the most recently domesticated species, which can produce meat and/or fur as human food and the material of high-grade textiles or can be bred and raised as pets [2, 3]. Also, rabbits are commonly used animal models for human biomedical diseases due to their characteristics: docile, non-aggressive, easy to handle, high reproductive [4]. Additionally, rabbits have been used as bioreactors for the production of pharmaceutical proteins such as polyclonal antibodies [5]. As an important animal closely related to human, the rabbit can not only provide human a variety of animal products, but also serves as a useful laboratory animal. The studies on rabbit genome and its second genome (the genome of microbes living on and in rabbit body) are helpful to apply rabbits as biomedical animal models and to reveal the molecular mechanism of genes related to rabbit diseases or economic traits. Therefore, it is necessary for us to understand rabbit host genome and its second genome.

With the rapid development of sequencing technologies, a mass of omics data on rabbit genome, transcriptome and microbiome have been generated and analyzed. Several remarkable progresses on the rabbit omics were worth mentioning. In 2005, a rabbit reference genome was first released from the NCBI genome database; and then recently in 2019 and 2020, another two high-quality reference genomes of rabbit were released (https://www.ncbi.nlm.nih.gov/genome/browse#!/eukaryotes/316/). In 2014, whole-genome sequencing of wild and domestic rabbits was performed to understand the genetic changes during rabbit domestication [6]. In 2015, transcriptome variations in peripheral blood mononuclear cells after in vitro stimulation by LPS or PMA-Ionomycin were investigated in the rabbit [7]. In 2016, hyperlipidemia-associated gene variations and expression patterns were revealed by whole-genome and transcriptome sequencing of three rabbit models [8]. In 2017, a transcriptome atlas of rabbit was revealed by PacBio single-molecule long-read sequencing, which provided a comprehensive set of reference transcripts and contributed to the improved annotation of rabbit genome [9]. And recently in 2019, liver transcriptome changes of Hyla rabbit in response to chronic heat stress were reported [10].

For rabbit microbiome, several works have been conducted by amplicon-based surveys of gut microbial communities in rabbits. Early in 2008, the bacterial community within the rabbit cecum was investigated [11]. In 2012, the cecal bacterial community of the rabbit was again studied using high throughput 16S rRNA gene V3-V4 amplicon sequencing [12]. In 2015, the gut microbiota of both hard and soft feces from Rex rabbits with high and low body weight were characterized using the Illumina MiSeq platform targeting the V4 region of the 16S rDNA [13]. In 2017, the impact of feed restriction and housing hygiene conditions on the cecal bacterial community of young rabbits was studied [14]. In 2018, rabbit microbiota changes throughout the intestinal tract were investigated, mainly including the microbiota of cecum and feces [15]. And in the same year, microbiome of total versus live bacteria in the gut of Rex rabbits was revealed [16]. In 2019, dynamic distribution of gallbladder microbiota in rabbit at different ages and health states were investigated [17]. In 2020, the dynamic distribution of gut microbiota in commercial Ira rabbits from weaning to finishing was investigated [18] and the relationship between the microbiota and average daily gain was uncovered via 16S rRNA gene sequencing [19]. More recently, bacterial microbiota composition along the gastrointestinal tract in New White Zealand rabbits was comprehensively characterized using next generation 16S rRNA gene sequencing [20].

However, previous rabbit microbial studies mostly focused on gut microbiota, especially the microbiota of cecum or feces. The microbiota in and around rabbit body, containing rabbit second genome, have not been all-round investigated. Here, we collected microbial samples from 12 body sites (skin, lung, uterus, mouth, stomach, duodenum, ileum, jejunum, colon, cecum, cecal appendix, rectum) of 4 healthy female adult Belgium gray rabbits. PCR and sequencing of V3-V4 region of 16S rRNA gene were carried out. The comparison of microbial communities and predicted microbial functions among rabbit whole body sites were performed. These data and results lay a foundation for the basic research of rabbit microbiome and further help to raise rabbits healthily and better support the application of rabbits as animal models.

## Materials and methods

### Experimental animals

The experimental animals were healthy female adult Belgium gray rabbits (n=4) of 12 weeks old, raised at a rabbit farm in Nanchang (28°50’34”N, 115°48’46”E), Jiangxi province, China. Rabbits were weaned at one month of age. These rabbits were under the same feeding procedures and management conditions. The rabbits were reared in single cages (dimensions: 40cm width × 60cm length × (45cm front height, 30cm back height)) of 12-cage with natural environmental conditions: the temperature and relative humidity ranged between 22-30 °C and 65-80%, respectively; the natural ventilation ranged between about 0.1-0.4 m^3^ s^-1^ and the natural photoperiod was about 14 h light per day at 30-100 lx. The rabbits were fed twice daily with regular adult rabbit pellets, which mainly contains the following ingredients: ground corn, alfalfa hay, barley malt root, soybean meal, calcium hydrogen phosphate, calcium carbonate, salt, multiple microelements, vitamins and amino acids, with 8.0% minimum crude fiber, 18.0% maximum crude fiber, 13.0% maximum crude ash, 16.0% minimum crude protein, 0.5% minimum calcium, 1.6% maximum calcium, 0.4% minimum phosphorus, 0.35% minimum methionine, and 14.0% maximum water (Table S1). Before slaughter, all rabbits were fasted for 24 h with free access to water.

### Sample collection

**Fig. 1:**
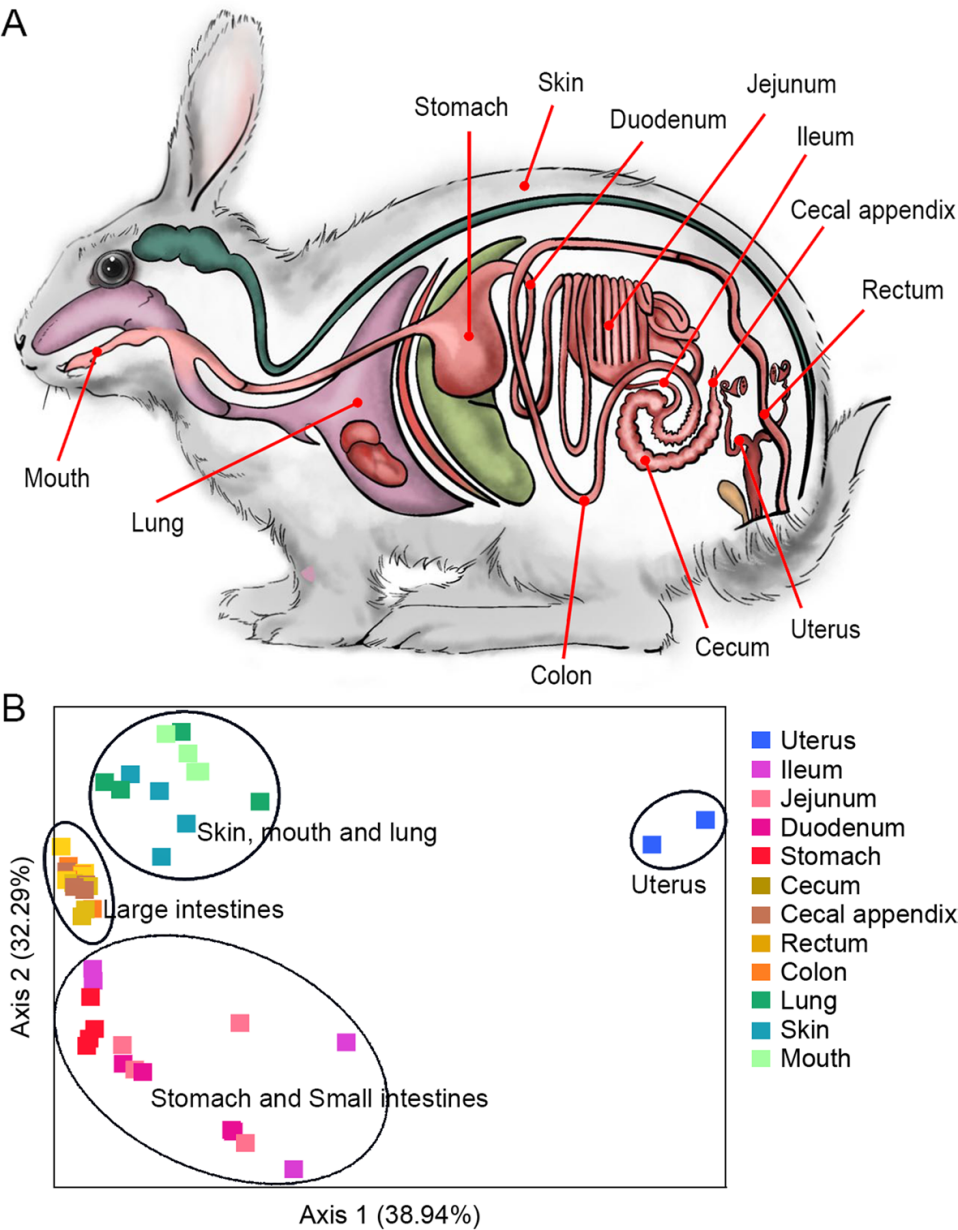
Sampling sites of microbial flora in rabbits (**A**) and the PCoA plot for microbiota across rabbit whole body based on the weighted UniFrac distances (**B**). These 46 samples roughly clustered into four groups: 1) the large intestine (Lin) group, 2) the stomach and small intestine (SSin) group, 3) the uterus (Uter) group and 4) the skin, mouth and lung (SML) group

Rabbits were sacrificed by the method of cervical dislocation. The carotid artery and jugular vein were immediately cut off to allow complete bleeding. Then, the microbial samples from 12 body sites were collected for each rabbit, including skin, uterus, mouth, lung, stomach, duodenum, ileum, jejunum, colon, cecum, cecal appendix and rectum (Fig. 1A).

The microbial samples from skin and mouth were collected using sterile swabs (Winner Medical, China). Before sample collection, the swabs were wetted with sterile PBS buffer (137 mM sodium chloride, 2.7 mM potassium chloride, 10 mM phosphate and 2 mM potassium phosphate). When collecting the samples from skin surface, the rabbit hair was pushed aside and the skin of the abdomen and on the back of the rabbit was wiped repeatedly with the wet swab; while collecting the mouth samples, the rabbit mouth was kept open and the mouth mucosa was wiped using the wet swab. The stem of the sampling swab was cut off, its head with the collected samples was placed into an antifreeze tube (LABSELECT, China), and then transferred and stored at -80°C. The microbial samples of uterus and lung were collected by flushing sterile PBS buffer. We flushed 1-2 ml PBS buffer into the uterus or lungs with a sterile syringe, and then gently kneaded it repeatedly for 2 minutes. The lavage fluid from uterus or lungs was collected into 15-ml sterile centrifuge tubes (LABSELECT, China), and refrigerated at +4°C. For each sample, 5-10 ml of lavage fluid was collected, and the pellet of lavage fluid was centrifuged at 4000 g for 30 min, 4°C and transferred into a 2 ml sterile centrifuge tube (LABSELECT, China). Then, the pellet was stored at -80°C until DNA extraction. Three sterile PBS buffer samples served as the negative controls. For the samples of stomach, small intestinal sites and large intestinal sites, we collected them by the same method. We first found the corresponding body sites and took a careful screening, then cut a 1-1.5 cm hole at these body sites, and collected their contents with sterilized spoons and placed them into sterile Eppendorf tubes (LABSELECT, China) marked by the body-site name. We used a new sterilized spoon at each gastrointestinal site per rabbit. The contents of stomach, duodenum, ileum, jejunum, colon, cecum, cecal appendix and rectum were stored at -80°C until use.

### DNA extraction, PCR amplification and sequencing

Microbial community genomic DNA was extracted from all 48 samples using the E.Z.N.A.® soil DNA Kit (Omega Bio-tek, Norcross, GA, U.S.) according to manufacturer’s instructions. The DNA extract was checked on 1% agarose gel, and DNA concentration and purity were determined with NanoDrop 2000 UV-vis spectrophotometer (Thermo Scientific, Wilmington, USA). The hypervariable region V3-V4 of the bacterial 16S rRNA gene were amplified with primer pairs 338F (5'-ACTCCTACGGGAGGCAGCAG-3') and 806R (5'-GGACTACHVGGGTWTCT AAT-3') (Sangon, China) by an ABI GeneAmp® 9700 PCR thermocycler (ABI, CA, USA).

The PCR amplification of 16S rRNA gene was performed as follows: initial denaturation at 95 °C for 3 min, followed by 29 cycles of denaturing at 95 °C for 30 sec, annealing at 55 °C for 30 sec and extension at 72 °C for 45 sec, and single extension at 72 °C for 10 min, and end at 4 °C. The PCR mixtures contain 5 × TransStart FastPfu buffer 4 μL, 2.5 mM dNTPs 2 μL, forward primer (5 μM) 0.8 μL, reverse primer (5 μM) 0.8 μL, TransStart FastPfu DNA Polymerase 0.4 μL, template DNA 10 ng, and finally ddH_2_O up to 20 μL. PCR reactions were performed in triplicate. The PCR product was extracted from 2% agarose gel and purified using the AxyPrep DNA Gel Extraction Kit (Axygen Biosciences, Union City, CA, USA) according to manufacturer’s instructions and quantified using Quantus™ Fluorometer (Promega, USA).

Purified amplicons were pooled in equimolar and paired-end sequenced (2 × 300) on an Illumina MiSeq platform (Illumina, San Diego, USA) according to the standard protocols by Majorbio Bio-Pharm Technology Co. Ltd. (Shanghai, China).

Three negative controls were extracted same as the above 48 samples following the same PCR and paired-end sequencing procedures.

### Bioinformatics analysis of sequencing data

The raw fastq files were filtered, denoised and merged and chimeric sequences were removed from amplicon sequence variants (ASVs) using the dada2 package [21] implemented in Qiime2-2019.07 [22]. Based on the sequence quality plots (Figure S1), forward and reverse reads were trimmed to 281 and 206 bp, respectively. The primer sequences were removed from all reads. The detailed command line was as follows: “qiime dada2 denoise-paired --i-demultiplexed-seqs paired-end-demux.qza --p-trim-left-f 20 --p-trim-left-r 20 --p-trunc-len-f 281 --p-trunc-len-r 206 --o-table table.qza --o-representative-sequences rep-seqs.qza --o-denoising-stats denoising-stats. qza”. After the filtration step, 40.9-92.5% (mean 70.8%) of the reads were left for further analysis (Table S2). Then the representative sequences and their numbers were identified and summarized using the feature-table function implemented in Qiime2-2019.07 [22]. The total number of representative sequences for the individual taxa was converted to a percentage, assuming the sum of all taxa in the individual samples to be 100%. Richness indices were calculated using the diversity function implemented in Qiime2-2019.07 [22].

The taxonomy of each 16S rRNA gene representative sequence was assigned by the SINA aligner and classification system [23], against the SILVA SSU database 138.1 [24]. Functional capacity of microbial community and function categorization based on the Kyoto encyclopedia of genes and genomes (KEGG) pathways was predicted using PICRUSt2 [25].

### Statistical analysis

Pairwise PERMANOVA analyses were performed to verify sample clustering in the PCoA using adonis.pair() implemented in the R package of EcolUtils [26]; the *P* values were adjusted by the method of Benjamini-Hochberg FDR. The difference significance of alpha diversity between pair-wise groups was tested by the Kruskal-Wallis test implemented in Qiime2-2019.07 [22]; the *P* values were corrected by the method of Benjamini-Hochberg FDR. For difference analyses of microbial abundance among the Lin, SSin, Uter and SML groups, the abundance data were first transformed into the rlog values using DESeq2 [27], then the ANOVA test with bonferroni correction was conducted using the statistical functions implemented in R. Comparisons of microbial abundances between pairwise different groups at the phylum and genus levels and comparisons of predicted abundances of KEGG orthologies or KEGG pathways between pairwise different body sites were performed using STAMP software [28]. For multiple test correction, Welch’s t test *P*-values were adjusted by the Benjamini-Hochberg FDR method. The 0.05 cutoff and the 0.01 cutoff were used as the significant threshold and the extremely significant threshold, respectively.

For the clustering of microbial communities at the genera level across rabbit whole body, the dendrograms of both the samples and features were generated using the “Heatmap plot” function implemented in STAMP software [28] with the parameters: clustering methods of “average neighbor (UPGMA)” and dendrogram clustering threshold of 0.75. While for difference of predicted functional capacities of microbiome among rabbit different body sites, the dendrograms were generated using the function Heatmap() of the R package “ComplexHeatmap” [29] with the default clustering parameters.

## Results

### Evaluation of the sequencing data quality

A summary of the sequencing data quality obtained in the present study is shown in Table S2. A total of 2,700,017 raw sequences were obtained, of which 2,663 (0.099%) were from negative controls. After the preliminary quality filtering, 2,403,784 sequences remained. Then, denoised quality filtering was conducted and 2,256,840 sequences were yielded, of which 2,041,066 sequences were the merged forward-reverse reads. Next, chimera removal was performed, 1,813,527 sequences remained. Finally, the relative number of passed reads after all the above steps was in the range of 40.9-92.5%. As a result, the sequence number of 46 samples were more than 20,000; two samples collected in rabbit uterus and all three negative controls contained less than 2,650 sequences. These two samples and three negative controls were excluded for the next analyses except where noted. Rarefaction curves were generated for all 46 samples. The graphs of observed ASVs and Shannon’s indexes showed each individual rarefaction curve reached a plateau (Fig. S2), indicating that the sequencing depth was close to saturation.

### ASVs identification and filtering

Based on the above qualified 1,813,527 sequences, we detected a total of 8,070 ASVs. These ASVs were classified into microbial taxa, and a total of 38 phyla and 494 genera were identified. After removing the ASVs with an average relative abundance of less than 0.025% and the ASVs only detected in one sample, we obtained 3,148 qualified ASVs, which occupied 93.8% of the total clean reads. In the next analyses of group comparison among the microbiome of different body sites, we used these 3,148 qualified ASVs. The summary of sequencing data and microbial structure identified in 12 rabbit body sites was shown in Table 1.

**Table 1:** Summary of microbial communities across multiple Rabbit body sites at different phylogenetic level

Group	Body site	Phyla number	Class number	Order number	Family number	Genus number	ASV number
Lin		15	21	38	62	130	2187
	Rectum	15	21	37	59	118	1721
	Cecum	15	20	36	57	116	1662
	Cecal appendix	15	21	37	59	122	1818
	Colon	15	21	37	57	115	1699
SSin		18	24	61	101	170	2049
	Jejunum	16	20	46	62	83	886
	Ileum	16	22	49	78	133	1417
	Duodenum	15	19	42	58	69	810
	Stomach	14	20	47	76	119	893
SML		22	31	62	114	191	804
	Skin	19	24	53	89	154	490
	Mouth	10	16	30	47	64	165
	Lung	19	27	51	81	119	396
Uter		16	20	45	70	93	332
	Uterus	16	20	45	70	93	332
In total		23	37	74	140	264	3148

Note: 1, the number indicates the total number of micbrobial communities in each body site or each group

### Microbial diversity at rabbit different body sites

We performed a principal coordinate analysis using the weighted UniFrac distances for these 46 samples at 12 different rabbit body sites. We found that these samples roughly clustered into four groups (Fig. 1B): 1) the large intestine (Lin) group, 2) the stomach and small intestine (SSin) group, 3) the uterus (Uter) group, and 4) the skin, mouth and lung (SML) group. The pairwise PERMANOVA analysis among these four groups verified the rationality of the clustering (Table S3). The Lin group included the microbial samples from colon, cecum, cecal appendix and rectum, which gathered closely; the SSin group contained the samples collected from stomach and small intestine (duodenum, ileum and jejunum), which was a loosely gathered group; the SML group consisted of the samples from mouth, lung and skin, which were separated from the above two groups of gastrointestinal tract at PCoA; the Uter group only contained the samples collected at the uterus, which formed a separate group keeping apart from the other three groups in the top left region of the PCoA plot. We merged Lin and SSin groups into the intestinal group, SML and Uter groups into the non-intestinal group. There were extremely significant difference between the intestinal and non-intestinal groups (Table S4).

**Fig. 2:**
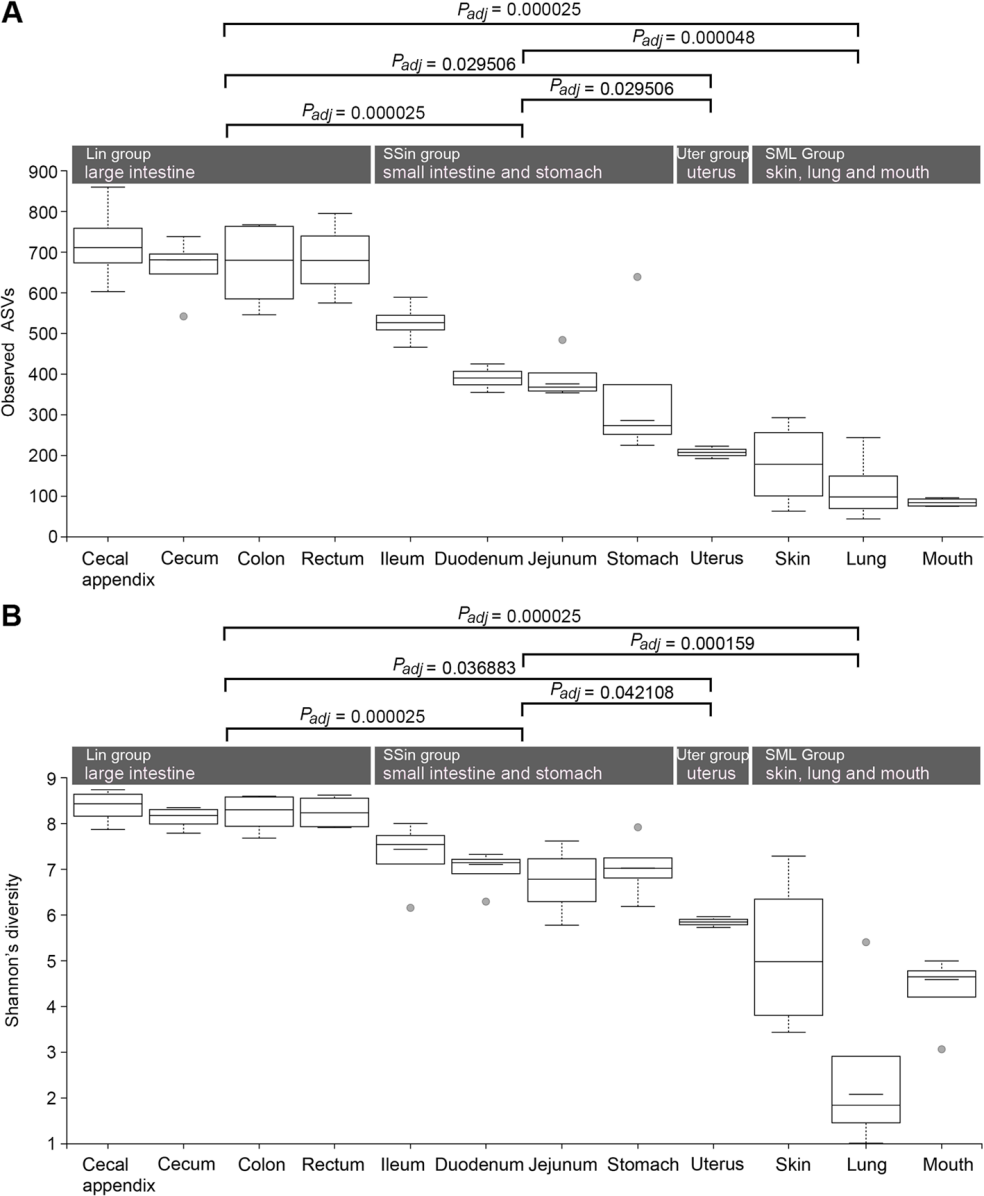
Differences in bacterial community diversity of observed ASVs (**A**) and Shannon’s diversity (**B**) among 12 rabbit body sites. The difference significance of alpha diversity between pair-wise groups was tested by the Kruskal-Wallis test implemented in Qiime2-2019.07. The *P* values were corrected by the method of Benjamini-Hochberg FDR

We compared the alpha-diversity of microbiota sampled from 12 rabbit body sites using the richness indices of observed species (Fig. 2A), the diversity index of Shannon (Fig. 2B), and pielou’s evenness index (Fig. S3). Among these 12 body sites, the diversity of microorganisms in cecal appendix was the highest, and those in mouth and lung were the smallest. If these samples were divided into 4 groups according to the PCoA clustering, the microbial diversity of the large intestine (Lin) was significantly higher than those of other three groups (*P* = 2.50 × 10^-5^, 2.95 × 10^-2^ and 2.50 × 10^-5^ when compared to groups SSin, Uter and SML, respectively, for observed ASVs; *P* = 2.50 × 10^-5^, 3.69 × 10^-2^ and 2.50 × 10^-5^ for Shannon’s index), and the one of small intestine and stomach (SSin) was significantly higher than those of uterus (Uter, *P* = 2.95 × 10^-2^ for observed ASVs and 4.21 × 10^-2^ for Shannon’s index) and skin, lung and mouth (SML, *P* = 4.80 × 10^-5^ for observed ASVs and *P* = 1.59 × 10^-4^ for Shannon’s index). If we divided the samples into the intestinal group (including Lin and SSin groups) and the non-intestinal group (including SML and Uter groups) (Fig. S4), the microbial diversity of the intestinal-group was significantly higher than that of the non-intestinal group (*P* = 1.39 × 10^-7^ for observed ASVs and 9.27 × 10^-7^ for Shannon’s index).

### Microbial community composition

**Fig. 3:**
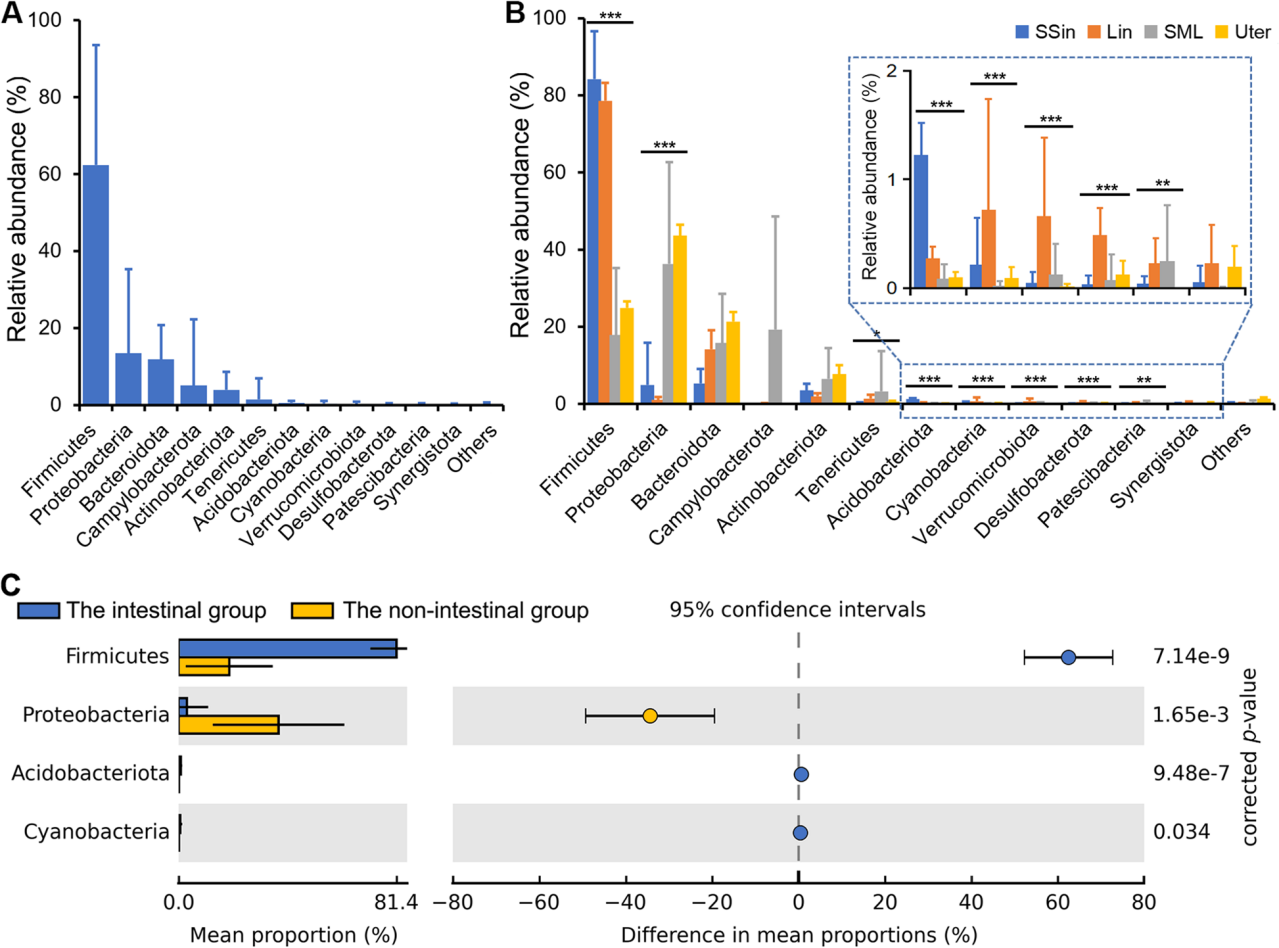
Overview and comparison of rabbit microbiota at the phylum level. **A** The phyla with relative abundance more than 0.05% were shown. The phyla with relative abundance < 0.05% are summarized as ‘Others’. **B** Comparison of twelve phyla with relative abundance more than 0.05% among the four groups. SSin represents the stomach and small intestinal group; Lin represents the large intestinal group; SML represents the skin, mouth and lung group; Uter represents the uterus group. Significant differences of phylum rlog-transformed abundance among the four groups were tested by the ANOVA analysis with the bonferroni correction (*, 0.01 ≤ *P* < 0.05; **, 0.001 ≤ *P* < 0.01; ***, *P* < 0.001). C. Four significantly different phyla between the intestinal group and the non-intestinal group, including two phyla with lower relative abundance < 0.05%. Welch’s t-test implemented in STAMP software was used; the *p*-values were corrected with Benjamini–Hochberg FDR method

Using the 3,148 qualified ASVs, a total of 23 phyla harboring 264 genera were detected. There were twelve phyla with a relative abundance of more than 0.1%, including *Firmicutes*, *Proteobacteria*, *Bacteroidota*, *Campylobacterota*, *Actinobacteriota*, *Tenericutes*, *Acidobacteriota*, *Cyanobacteria*, *Verrucomicrobiota*, *Desulfobacterota*, *Patescibacteria* and *Synergistota*. The total relative abundance of other phyla was less than 0.3%. Among these twelve phyla with higher relative abundance, *Firmicutes* (62.4%), *Proteobacteria* (13.4%), *Bacteroidota* (11.8%) and *Campylobacterota* (5.1%) were the most predominant (Fig. 3A), and eight phyla of *Firmicutes* (*P* = 6.42 × 10^-13^, ANOVA test for the rlog-transformed abundance values with bonferroni correction), *Proteobacteria* (*P* = 2.29 × 10^-6^), *Tenericutes* (*P* = 4.99 × 10^-2^), *Acidobacteriota* (*P* = 1.77 × 10^-8^), *Cyanobacteria* (*P* = 5.82 × 10^-6^), *Verrucomicrobia* (*P* = 2.44 × 10^-5^), *Desulfobacterota* (*P* = 1.11 × 10^-9^), and *Patescibacteria* (*P* = 2.90 × 10^-3^) showed significant differences among the Lin, SSin, SML and Uter groups (Fig. 3B).

We compared the microbial community composition between different pairwise groups at the phylum level. Interestingly, we observed that both the most abundant phyla (*Firmicutes* and *Proteobacteria*) showed significant differences between the intestinal group containing the Lin and SSin groups and the non-intestinal group involving the SML and Uter groups (Fig. 3C). The relative abundance of *Firmicutes* in the SSin (84.2%) and Lin (78.6%) groups was far higher than that in the SML (17.9%) and Uter (24.9%) groups. On the contrary, the relative abundance of *Proteobacteria* in the SSin (4.9%) and Lin (1.0%) groups was much lower than that in the SML (36.3%) and Uter (43.6%) groups. In addition, two other phyla *Acidobacteriota* and *Cyanobacteria* with low abundance were also significantly different between the intestinal and non-intestinal groups (Fig. 3C).

When the SSin group was compared with the Lin group, there was no difference in two phyla of *Firmicutes* and *Proteobacteria*, which had high abundance, and also no difference in 12 low-abundance phyla, but there were significant differences in the phyla *Bacteroidota*, which had high abundance, and seven phyla with medium abundance including *Actinobacteriota*, *Tenericutes*, *Acidobacteriota*, *Verrucomicrobiota*, *Desulfobacterota*, *Patescibacteria* and *Crenarchaeota* (Fig. S5A). When the SSin group was compared with the SML group, the two phyla of *Firmicutes* and *Proteobacteria* with high abundance and another phylum *Acidobacteriota* with medium abundance existed significant differences (Fig. S5B). These three phyla also showed significant differences in the comparison between the SSin and Uter groups (Fig. S5C) and the comparison between the Lin and SML groups (Fig. S5D). Besides, two low-abundance phyla *Crenarchaeota* and *Chloroflexi* existed differences between the SSin and Uter groups, while two low-abundance phyla *Desulfobacterota* and *Verruco-microbiota* existed differences between the Lin and SML groups. When the Lin group was compared with the Uter group, there was significant difference in the highest abundance phylum *Firmicutes*. Besides, the two phyla *Verrucomicrobia* and *Patesci-bacteria* existed significant differences (Fig. S5E). When the SML group was compared with the Uter group, only one low-abundance phylum *Crenarchaeota* was significantly different (Fig. S5F).

We further examined the microbial communities at the genus level. A total of 198 classified genera and 66 unclassified genera were detected in these 46 samples from 12 body sites. The relative abundance of 198 classified genera was 47.6%, while the one of 66 unclassified genera was 52.4%. There were 24 genera with relative abundance more than 1.0%. Among them, the unclassified genera annotated to the order Clostridiales (belonging to the Phyla *Firmicutes*) was the richest, accounting for 29.0% and containing 733 ASVs; the second and third highest genera was the unclassified genera belonging to the family Eubacteriaceae (*Firmicutes*) with a relative abundance of 4.6% and NK4A214 group (*Firmicutes*) with a relative abundance of 4.1%. In the Lin group, four genera of NK4A214 group (*Firmicutes*), Ruminococcus (*Firmicutes*), two unclassified genera belonging to the order Clostridiales (*Firmicutes*) and the family Muribaculaceae (*Bacteroidota*) were the predominant genera with relative abundance more than 5.0%; in the SSin group, the genus of Faecalibaculum and two unclassified genera belonging to the order Clostridiales and the family Eubacteriaceae, which were all belonging to the phyla *Firmicutes*, were the predominant genera with relative abundance more than 5.0%; in the Uter group, six genera of Stenotrophomonas (*Proteobacteria*), Ralstonia (*Proteobacteria*), Acineto-bacter (*Proteobacteria*), Bacteroides (*Bacteroidota*), and the unclassified genera belonging to the orders Clostridiales (*Firmicutes*) and Bacteroidales (*Bacteroidota*), were the predominant genera with relative abundance more than 5.0%; in the SML group, seven genera of Actinobacillus (*Proteobacteria*), Bordetella (*Proteobacteria*), Helicobacter (*Campylobacterota*), Aliarcobacter (*Campylobacterota*), Bacteroidota (*Filobacterium*), Bergeyella (*Bacteroidota*) and the unclassified genus belonging to the family Pasteurellaceae (*Proteobacteria*), were the predominant genera with relative abundance more than 5.0%.

We also compared the microbial community composition between different pairwise groups at the genus level (Table S5). We found that the number of different genera of the Lin group were the most, when compared to other three groups (SSin, SML and Uter groups). There were 82, 70 and 64 different genera in the comparison of the Lin group with the SML, SSin and Uter groups, respectively. While a few of different genera were observed in pairwise comparisons among the SML, SSin and Uter groups. There were only 5 different genera between the SSin and SML groups, 8 between the SSin and Uter groups, and 5 between the SML and Uter groups. Further, we found that the relative abundances of 46 genera were higher in the Lin group than those in the other groups (Table S5). The relative abundances of Bacteroides (*Bacteroidota*), Acidovorax (*Proteobacteria*) and an unclassified genus belonging to the order Bacteroidales (*Bacteroidota*) in the Uter group was higher than those in other groups. Faecalibaculum (*Firmicutes*), and three unclassified genera belonging to the family Eubacteriaceae (*Firmicutes*), the orders Clostridiales (*Firmicutes*) and Acidimicrobiales (*Acidobacteriota*), respectively, had higher relative abundance in the SSin group than in other groups.

**Fig. 4:**
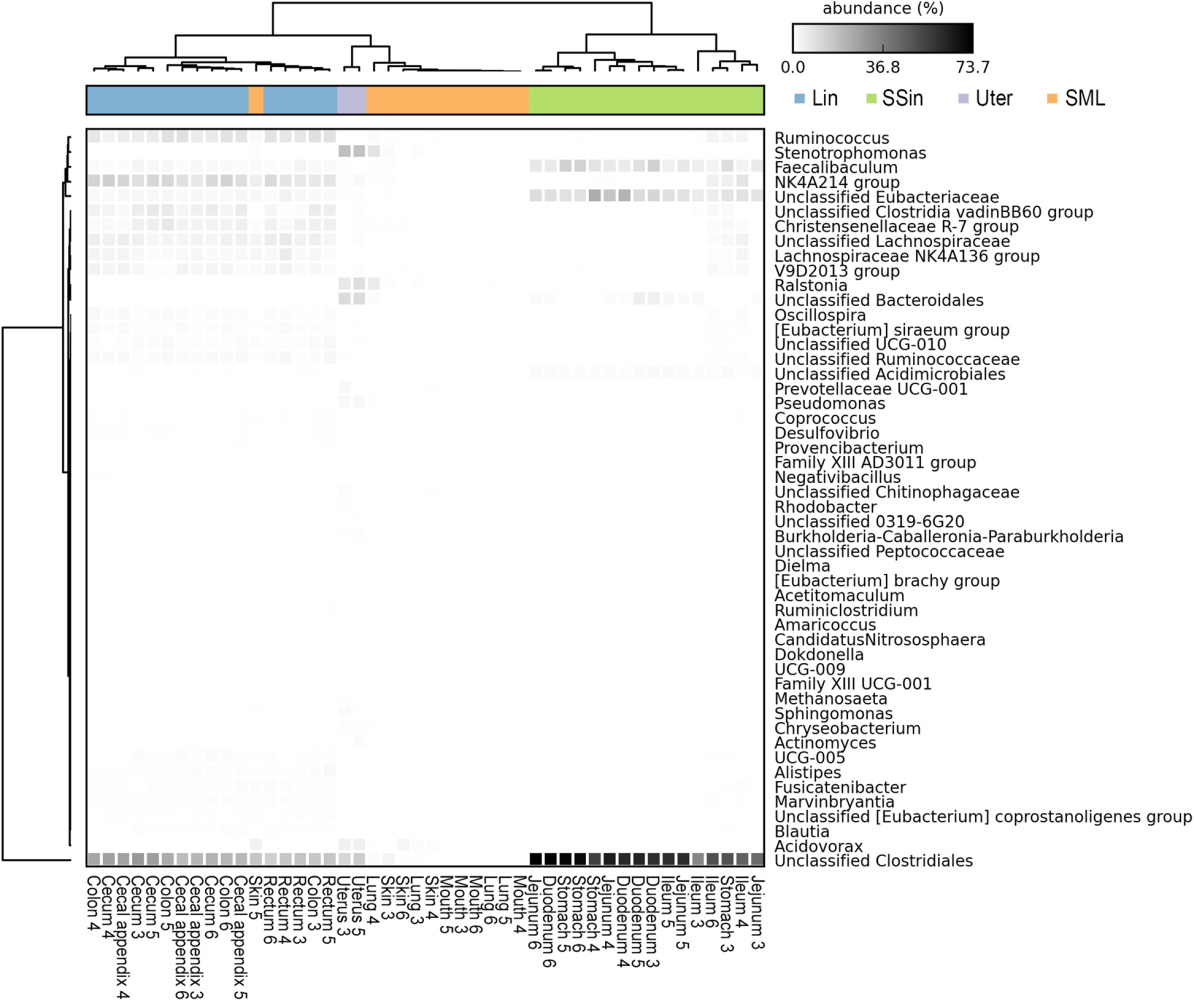
Heatmap and clustering of microbial communities at the genera level across rabbit whole body. The used bacteria contain 54 top abundance genera. This clustering results are generally consistent to the PCoA clustering using all ASVs. The samples were clustered into four groups: large intestinal (Lin), stomach and small intestinal (SSin), uterus (Uter), and skin, mouth and lung (SML) groups. A skin microbial sample was clustered into the large intestinal groups possibly due to fecal contamination of the individual’s skin

Using the top 50 genera with the highest relative abundance, we built a hierarchical clustering heatmap for the 46 samples (Fig. 4). Generally, these microbial samples were also clustered into four groups, including the SSin, Lin, SML and Uter groups. The clustering results were consistent to those generated from their OUTs, except one sample from the skin of individual No. 5. This sample was gathered into the large intestine group, indicating that this sample from skin might be contaminated by feces.

### Core and unique microbiome

**Fig. 5:**
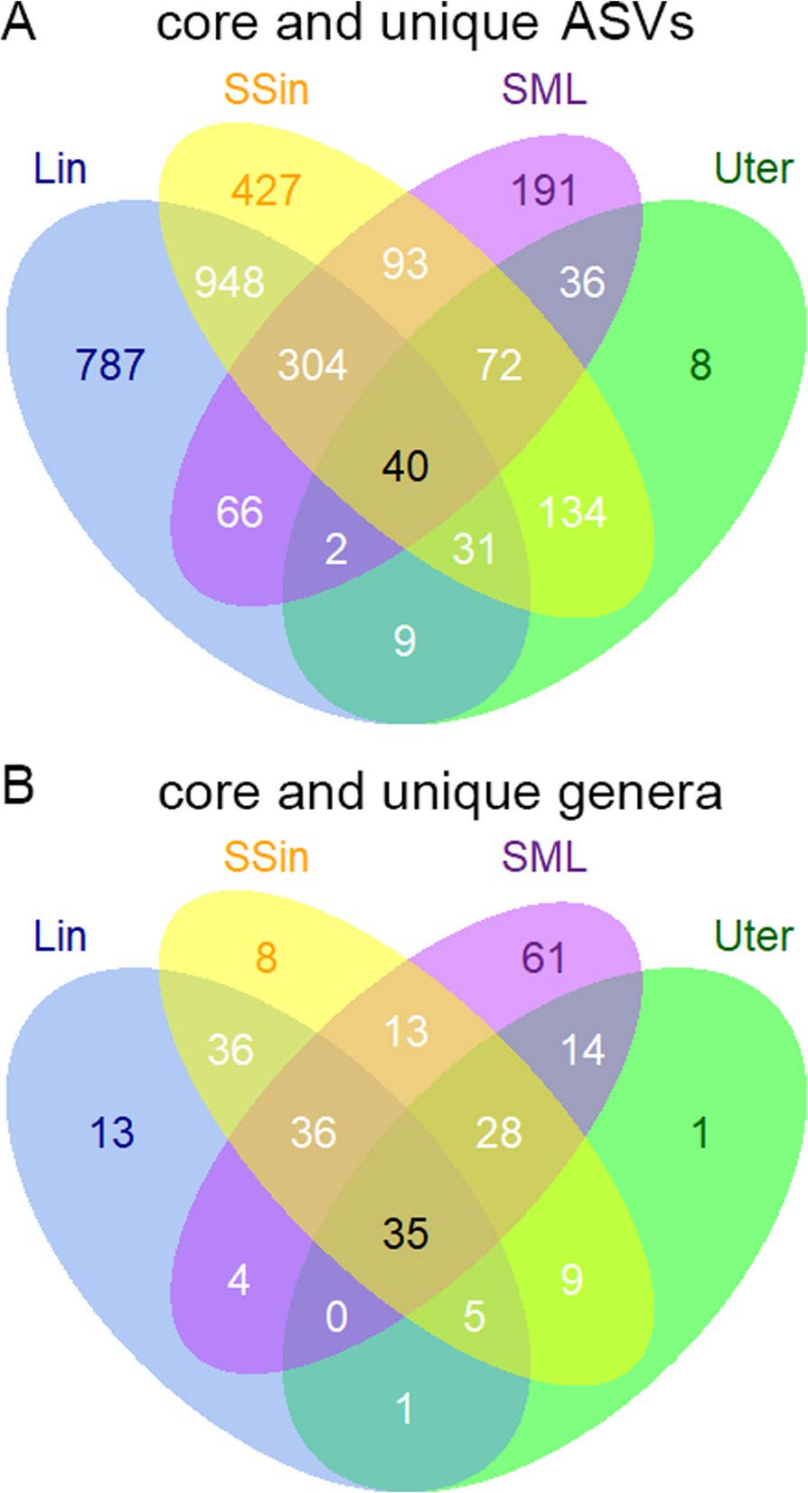
Venn graphs of core and unique ASVs (**A**) and genera (**B**) among the Lin, SSin, SML and Uter groups

Among the 3,148 ASVs, we found only 40 ASVs were shared by all the four groups of Lin, SSin, SML and Uter (Fig. 5A). These 40 ASVs were defined as core microbiome of rabbit bodies and were annotated to 4 phyla and 21 genera. Interestingly, the Lin and SSin groups shared the greatest number of ASVs (1,323), far more than the sharing number of ASVs in comparison of other pair-wise groups (Lin vs SML: 412; SSin vs SML: 509; SSin vs Uter: 277; SML vs Uter: 150); while the Lin and Uter groups shared the lowest number of ASVs (82), far less than the sharing number of ASVs in comparison of other pair-wise groups. We defined the ASVs only detected in one specific group as its unique microbiome. There were 787 unique ASVs (annotated to 11 phyla 73 genera) in the Lin group, 427 (12 phyla 35 genera) in the Sin group, 191 (16 phyla 90 genera) in the SML group and only 8 (7 phyla 8 genera) in the Uter group. When we performed the same analysis at the genus level, we found that 35 genera (annotated to 6 phyla) were shared among the four groups (Fig. 5B), which were the core microbiome in the whole body of rabbit. Only one genus was endemic in the Uter group, 8 in the SSin group, 13 in the Lin group, and the greatest number of unique genera in the SML group, the genera number was 61. Among these 61 genera, 27 genera were only found in skin, 4 were specific in oral cavity and 4 were only detected in lung.

In summary, there were few sharing microbes among all the rabbit different bodies. The SML group, especially the skin, contains the greatest number of unique microbes.

### Functional prediction and their differences among different groups

To compare the potential functional capacities of microbiome among rabbit different body sites, functional profiles of microbiome were predicted based on 16S rRNA gene sequencing data.

We detected 273 KEGG pathways showing significant difference with FDR adjusted *P* < 0.05 between the intestinal and non-intestinal groups (Table S6). 169 out of 273 KEGG pathways were higher in the non-intestinal groups, among which Superpathway of pyrimidine deoxyribonucleotides de novo biosynthesis (PWY-7211), fatty acid elongation -- saturated (FASYN-ELONG-PWY), Superpathway of tetrahy- drofolate biosynthesis and salvage (FOLSYN-PWY), Pyrimidine deoxyribonucleo- tides de novo biosynthesis I (PWY-7184), Inosine-5'-phosphate biosynthesis III (PWY-7234), superpathway of guanosine nucleotides de novo biosynthesis I (PWY-7228), polyisoprenoid biosynthesis (*E. coli*, POLYISOPRENSYN-PWY), anhydromuro- peptides recycling (PWY0-1261), pyrimidine deoxyribonucleotide phosphorylation (PWY-7197), superpathway of tetrahydrofolate biosynthesis (PWY-6612) were the top 10 pathways with the most significant *P* values. While 104 KEGG pathways were higher in the intestinal groups, among which cob(II)yrinate a,c-diamide biosynthesis I (early cobalt insertion, PWY-7377), tRNA charging (TRNA-CHARGING-PWY), purine ribonucleosides degradation (PWY0-1296), superpathway of aromatic amino acid biosynthesis (COMPLETE-ARO-PWY), pentose phosphate pathway (non-oxidative branch, NONOXIPENT-PWY), chorismate biosynthesis I (ARO-PWY), superpathway of pyrimidine deoxyribonucleosides degradation (PWY0-1298), glycolysis III (from glucose, ANAGLYCOLYSIS-PWY), superpathway of purine deoxyribonucleosides degradation (PWY0-1297), chorismate biosynthesis from 3-dehydroquinate (PWY-6163) were the top 10 pathways with the most significant *P* values.

**Fig. 6:**
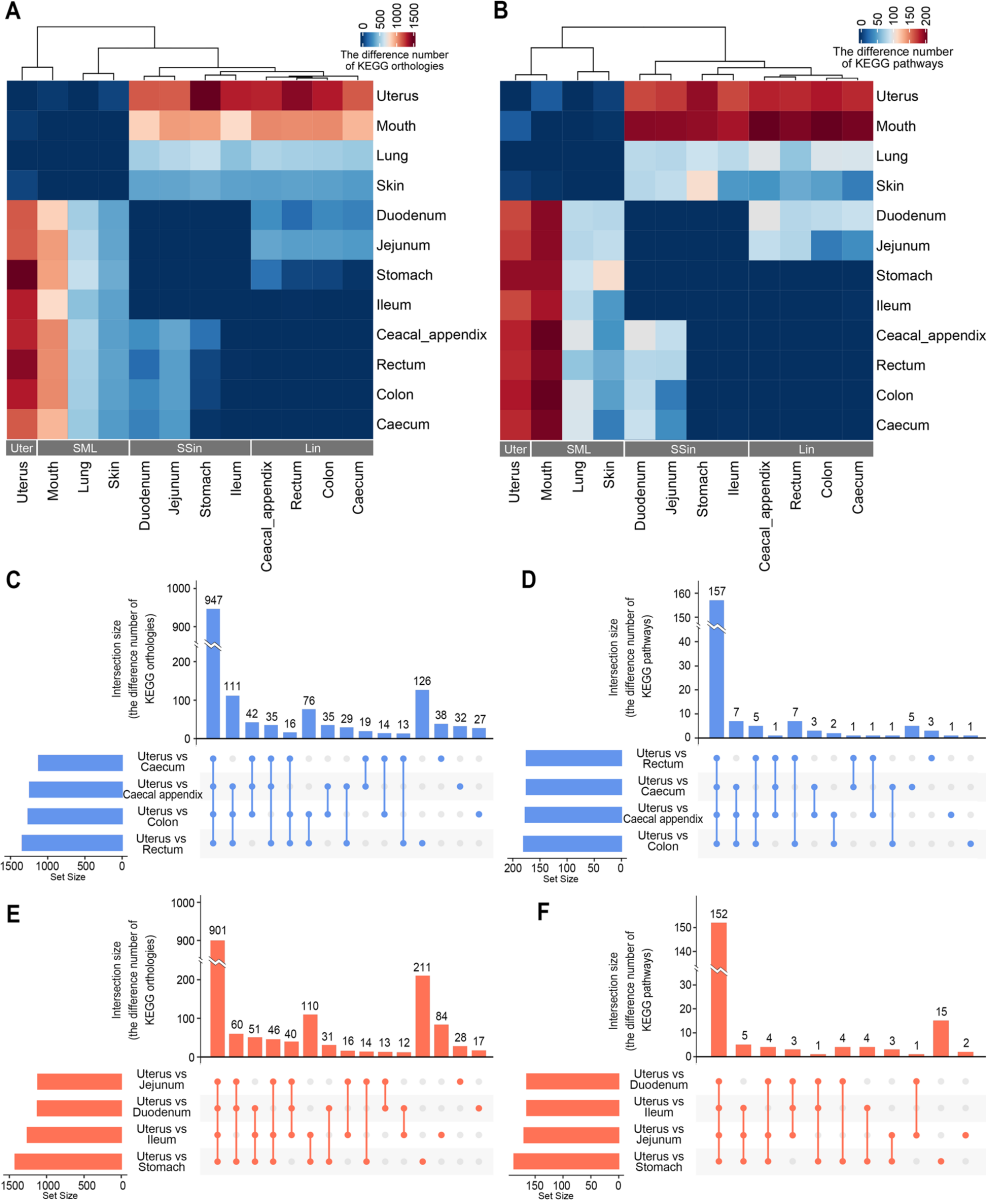
Difference of predicted functional capacities of microbiome among rabbit different body sites. **A** Heatmap for pair-wise differences of KEGG orthologies across 12 rabbit body sites. **B** Heatmap for pair-wise differences of KEGG pathways across 12 rabbit body sites. **C** Upset graph for the differences of KEGG orthologies between the uterus and large intestinal sites. **D** Upset graph for the differences of KEGG pathways between uterus and large intestinal sites. **E** Upset graph for the differences of KEGG orthologies between the uterus and stomach and between the uterus and small intestinal sites. **F** Upset graph for the differences of KEGG pathways between the uterus and stomach and between the uterus and small intestinal sites

Moreover, we compared the potential functional capacities of microbiome between the sample groups from different body sites (Fig. 6 and Table S7). We found that there was almost no difference in the comparison of the potential microbial functional capacities of the samples at each body sites within each Lin, SSin and SML group. For examples, within the Lin group, the potential microbial functional capacities of ceacal appendix, ceacum, rectum and colon had no difference when compared to each other; within the SSin group, those of stomach, duodenum, jejunum and ileum had no difference when compared to each other. However, there were many significant differences in the microbial functional capacities when compared the samples among the Lin, SSin, SML and Uter groups (Fig. 6A and B). The difference number of KEGG Orthologies (KOs) between gastrointestinal tract (including stomach, small intestinal and large intestinal sites) and uterus (1240.3 ± 113.1) was the largest, followed by the difference KO number between gastrointestinal tract and mouth (933.6 ± 89.9) and that between gastrointestinal tract and lung (526.9 ± 41.7). For the predicted KEGG pathways, the difference number between gastrointestinal tract and mouth (192.9 ± 6.5) was the largest, followed by those between gastrointestinal tract and uterus (174.4 ± 7.7) and between gastrointestinal tract and lung (84.4 ± 9.3). Notably, there were almost no difference between ileum (belonging to the SSin group) and large intestinal sites, possibly due to ileum is the nearest site to the large intestine. And there were little differences of KOs and no differences of KEGG pathways between stomach and large intestinal sites, possibly due to rabbit coprophagy, the behaviour of consuming soft feces.

The difference of predicted microbial functions between uterus and mouth was small and both clustered together, while the differences between lung and skin, between duodenum and jejunum, and between stomach and ileum were small, too. However, the function of microorganism in uterus and mouth were much different from those in the gastrointestinal sites. We further examined the differences comparing the microbial function in uterus to those in the SSin and Lin groups, including stomach, three small intestinal and four large intestinal sites. A total of 1,757 KEGG orthologies and 207 KEGG pathways showed significant difference between the uterus and the large intestines, while comparing the uterus to the SSin group, a total of 1,183 KEGG orthologies and 180 KEGG pathways with significant difference were found. We further observed 947 common differential microbial KOs and 157 common differential KEGG pathways when comparing uterus to the four large intestinal sites (Fig. 6C and D), and 901 common microbial KOs and 152 common differential KEGG pathways between uterus and the four SSin sites (Fig. 6E and F).

The top ten significant pathways in comparisons of uterus to the large intestines were phosphatidylglycerol biosynthesis II (non-plastidic), phosphatidylglycerol biosynthesis I (plastidic), superpathway of adenosine nucleotides de novo biosynthesis I, starch degradation V, glycogen biosynthesis I (from ADP-D-Glucose), superpathway of L-aspartate and L-asparagine biosynthesis, L-leucine degradation I, superpathway of L-methionine biosynthesis (by sulfhydrylation), superpathway of glycolysis and Entner-Doudoroff, and superpathway of sulfate assimilation and cysteine biosynthesis. Among these pathways, phosphatidylglycerol biosynthesis II (non-plastidic), phosphatidylglycerol biosynthesis I (plastidic), superpathway of adenosine nucleotides de novo biosynthesis I, starch degradation V, glycogen biosynthesis I (from ADP-D-Glucose), superpathway of L-aspartate and L-asparagine biosynthesis were with higher abundance in the large intestines than in the uterus; on the contrary, L-leucine degradation I, superpathway of L-methionine biosynthesis (by sulfhydrylation), superpathway of glycolysis and Entner-Doudoroff, and superpathway of sulfate assimilation and cysteine biosynthesis were with higher abundance in the uterus.

## Discussion

The human microbiome is composed of the microbes living in and on the human body, which has been treated as human second genome and is a source of genetic diversity, a modifier of disease, an essential component of immunity, and a functional entity that influences metabolism and modulates drug interactions [30]. Integration of microbial genomic data sets will be helpful in analyzing genetic variation and risk of human disease. The same is true of the microbiome of other mammals, including commonly used experimental or livestock animals such as rabbits. Therefore, it is necessary performing a comprehensive survey of the microbiota living on and in mammal body. However, except for the systematic investigation of human microbiome, few comprehensive surveys of microbiome across multiple body sites were carried out on mammals. Most studies have focused on local microbes in specific parts, such as gut microbes [16, 20, 31] or respiratory tract microbes [32, 33]. Rabbits share close relationship with humans, which can produce useful animal products for humans or serve as pets or biomedical animal models [2, 3]. Here we employed 16S rRNA gene sequencing technology to comprehensively investigate the rabbit microbiome by surveying the microbial communities across the whole bodies of 4 healthy female Belgium gray rabbits, including 12 body sites: skin, lung, uterus, mouth, stomach, duodenum, ileum, jejunum, colon, cecum, cecal appendix and rectum.

In our present study, the microbes across whole body of healthy rabbit could roughly cluster into four groups: Lin (large intestine), SSin (Stomach and small intestine), Uter (uterus), and SML (skin, mouth, lung) groups. In humans, the microbes across multiple body sites of healthy individuals clustered into four major body habitat groups [34], including oral, skin, gut and vaginal groups. As for the diversity of microbiome across whole body sites, we found that the phenomena similar to those in humans [30]: the gut microbiome shows low diversity at higher phylogenetic levels (for example, at the phylum level) but contains great diversity at lower phylogenetic levels (here at the ASV level) (Table 1). We could observe that the phyla and class number of Lin microbes are 15 and 21 respectively, which were smaller than those (22 and 31) of SML microbes, while the number of ASVs (2187) in the Lin group was much larger than that (804) of the SML group. We also found that the microbiota of different groups in rabbits were quite different. There were 40 shared ASVs, only accounting for 1.27% of the total ASVs. The huge differences of microbial communities in different body sites were similar as those in humans [35].

Although the microbial diversities in various parts of the large intestine were all high, we found that the microbial communities were relatively stable. On the PCoA plot based on the weighted UniFrac distances (Fig. 1B), the microbiota in cecum, cecal appendix, colon and rectum were clustered in a very small area; in addition, the microbial prediction function analyses (Fig. 6A and B) also showed there were little difference among them in different parts of the large intestine. Both of the results could reflect the stability of microbial community in various parts of the large intestine. According to the clustering degree of principal coordinates, we found the microbial communities in ileum varies most largely among four tested samples, followed by the microbiota in jejunum and lung. The microbial composition in uterus was significantly different from those in other body sites.

In the microbial communities of rabbit whole body, *Firmicutes*, *Proteobacteria* and *Bacteroidota* are the most abundant three phyla with relative abundance of more than 10%. Their distribution in each part was also significantly different. *Firmicutes* was significantly more in the intestine (81.4%) than in the non-intestine (18.9%), whereas *Proteobacteria* was significantly less in the intestine (3.0%) than in the non-intestine (37.3%). *Bacteroidota* was smallest in the small intestine (5.3%) than other groups including the large intestine (14.2%), SML (15.8%), Uter (21.3%) groups. In the *Firmicutes* phylum, Clostridia was the most abundant microbial class followed by Bacilli.

Previous studies on rabbit microbial communities focused on gut microbiota mainly sampled from cecum and/or feces [11, 12, 14, 15], which were equivalent to our samples from large intestine. Thus, we compared our results of rabbit microbiota in large intestine with previous studies. We found that the main phyla (*Firmicutes* and *Bacteroidota*) of large intestinal microorganisms were the same as previously reported [11, 12, 14, 15], and *Firmicutes* shows a large dominance. However, the relative abundance of the two phyla was different from previous studies [11, 12] to some extent. In our study, the abundance of *Firmicutes* in the large intestine was 78.6%, *Bacteroidota* was 14.2%, followed by *Actinobacteriata* (2.0%) and *Tenericute* (1.4%). Our results are very different from the two earliest studies [11, 12], which reported that the abundance of *Firmicutes* was more than 90% and that of *Bacteroidota* was about 4.0%. The source of this difference may be due to the update of the database. Previous analysis did not separate *Tenericutes* from *Firmicutes*. Then in the study of Combes et al. [14], *Firmicutes* represented 83% of the total DNA sequences, *Bacteroidota* represented 5.8%, followed by *Proteobacteria* (0.58%) and *Actinobacteria* (0.37%). Recently in 2018, Velasco-Galilea et al. [15] found phyla *Firmicutes* (76.4%), *Tenericutes* (7.8%), and *Bacteroidota* (7.4%) dominate the microbial diversity of rabbit caecal and faecal microbiota. At the phyla level, the relative abundance of *Firmicutes* is very similar to our results, but large differences existed for *Bacteroidota* and *Tenericutes*. The abundance of *Bacteroidota* was about two times as high as that of them, and the abundance of *Tenericutes* was about one fifth of them. The source of this difference may be related to the feeding conditions of rabbits (such as nutrient composition of feed and living environment) or different rabbit breeds. Our results of microbial abundance are far from those of Zeng et al. [13]. They used feces as intestinal microorganisms and found that the abundance of *Firmicutes* was 33.9-42.8%, *Bacteroidota* was 33.2-43.7%, *Tenericutes* was 1.8-5.6%. This may be related to the different research methods, such as sequencing region of 16S rRNA gene and taxonomic annotation.

In our present study, we have collected 4 microbial samples from the uterus of 4 female rabbits. However, due to low biomass and small sequence data, two samples did not pass the quality control and were filtered out. Therefore, only two replicates were included for the uterus comparisons. Using these two retained samples, we found that there were four dominant phyla in rabbit uterus, from more to less: *Proteobacteria* (43.6%), *Firmicutes* (24.9%), *Bacteroidota* (21.3%) and *Actinobacterita* (7.7%). Similarly, a recent study on human endometrial microbiota showed that the most abundant phylum in endometrium of the health infertile females without intrauterine lesion was *Proteobacteria*, which accounted for 68.11%, followed by *Firmicutes* (16.01%), *Bacteroidota* (8.38%) and *Actinobacteria* (5.73%) [36]. Meanwhile, we found that among the top 10 bacterial genera, three genera were observed in both the human study and our present rabbit study, including *Stenotrophomonas*, *Acinetobacter* and *Pseudomonas*. Early in 2016, Fang et al. investigated the relation of endometrial polyps to local microbiota by the 16S rRNA gene sequencing analysis of transcervical uterine swabs [37]. They also found that *Proteobacteria* (45.3%), *Firmicutes* (35.1%), *Actinobacteria* (8.9%) and *Bacteroidota* (~2.5%) were the top four bacterial taxa in the intrauterine microbial communities at the phylum level. In addition, *Lactobacillus* was often detected as a predominant genus in the uterine microbial community [38]. Here in our study, this genus was also found with the relative abundance of 0.97% in the uterus of health female rabbit.

In the prediction of the potential function of microbiome, the microbial function of the large intestines had little or no differences with those of stomach and ileum, which was roughly consistent with the clustering results of ASVs. We could observe that four stomach samples and two ileum samples shared close distance with the large intestines at the first axis of the PCoA plot (Fig. 1B). However, in human [39] and other mammals like pig [40], the microbial community of stomach is very different compared to that of colon, cecum or feces due to the high acidic environment of the gastric mucosa. It was possibly explained by that: 1) since the ileum is closest to large intestines, their microbial function was similar showing no significant difference with the one of large intestines; 2) rabbit’s coprophagy of consuming soft feces resulted in little differences of KOs and no differences of KEGG pathways between stomach and large intestinal sites. Rabbit coprophagy was first reported in the scientific literature in 1882 [41]. Rabbits produced 2 types of feces, soft mucous-covered feces during the night and hard dry pellet-like feces in the daytime; and rabbits took soft feces directly from their anus and swallowed them like pills [41, 42]. Coprophagy is of significance in rabbits because soft-feces consumption is conducive to the establishment of a nutritious intestinal microflora in rabbits and the soft feces supply rabbits with nitrogen, protein, sulfur and vitamins. Previous study showed that rabbit soft feces are similar in composition of cecal contents and have had an extended period of time in the cecum [43]. The elimination of soft feces is preceded by an increased activity of the distal part of the proximal colon, moving its contents, in 2 to 3 hours, toward the rectum [44]. In our present study, we found that there was less difference number of microbial KEGG orthologies between stomach and cecum than those between stomach and other large intestines, and the difference numbers of microbial KEGG orthologies between stomach and cecum, between stomach and colon, and between stomach and rectum was gradually increasing along the large intestinal tract (Fig. 6A and Table S7), which suggested that our results were well consistent to the progresses of soft feces formation and elimination.

It is worth mentioning that the microbial function of the uterus and the large intestine is the most different. A total of 1,757 KEGG orthologies and 207 KEGG pathways showed significant difference between the uterus and the large intestines. Notably, two top pathways of phosphatidylglycerol biosynthesis showed significant differences between the uterus and the large intestines, and their relative abundances were higher in the large intestines than in the uterus. Phosphatidylglycerol is a ubiquitous phospholipid in the biological membranes of prokaryotes [45] and eukaryotes [46]. The phosphatidyl-glycerol biosynthesis is required for the development of embryos and normal membrane structures of chloroplasts and mitochondria [47]. The phosphatidylglycerol biosynthesis could be inhibited with sulfhydryl poisons [48]. Interestingly, among the top different pathways, superpathway of L-methionine biosynthesis (by sulfhydrylation) and superpathway of sulfate assimilation and cysteine biosynthesis were with significantly higher abundance in the uterus than in the large intestines. It is reported that sulfur amino acid metabolism influences reproductive physiology [49], and transsulfuration activity can support cell growth [50]. Recent study in mice showed that myometrial sulfur amino acid metabolism might regulate uterine redox homeostasis and contribute to the source and metabolism of myometrial cysteine during estrus and pregnancy [51]. Therefore, based on our findings, we could make a hypothesis that the microbes in the uterus might be helpful in the biosynthesis of L-methionine and cysteine and play roles in keeping uterine redox homeostasis in rabbit.

There were several major limitations in our present study. First, we did not include microbial samples from various body sites of male individuals. Our results could not reflect the changes of microorganisms between rabbit sexes. Second, in this study only 16S rRNA gene sequencing was applied. There was little information annotated at the species level, and large part of genera, families or orders were not annotated and grouped into unclassified microbes. This limitation would be solved by the improvement of microbial annotation database or application of metagenomic sequencing. Third, only two uterine samples passed quality control and were used in the subsequent comparisons. Although some previous studies in human or other mammalian uterine microbiome supported the similar findings as found in our present study, it is still necessary to collect more valid uterine samples in our future research. So that we will gain more reliable results. In addition, we used PICRUSt2 to predict the function of microorganisms. These functions need to be further verified by biological experiments.

## Conclusions

In this study, the microbiota across rabbit whole body was investigated via 16S rRNA gene amplicon sequencing. *Firmicutes*, *Proteobacteria* and *Bacteroidota* were the most predominant phyla. The relative abundance of *Firmicutes* in the intestinal tract was significantly higher than that in the non-intestinal site, while *Proteobacteria* was significantly higher in the non-intestinal site. The function of microorganism in uterus and mouth were the most different from those in the gastrointestinal sites; rabbit’s coprophagy of consuming soft feces possibly resulted in little differences of microbial function between stomach and large intestinal sites.

## Supplementary Information

**Additional file 1: Table S1.** Rabbit food ingredients and guaranteed analysis. **Table S2.** Detailed information of the microbial data. **Table S3.** Pairwise PERMANOVA analysis for the sample clustering^1^. **Table S4.** Pairwise PERMANOVA analysis for the clustering of intestinal and non-intestinal groups^1^. **Table S5.** Comparison of microbiome among four different groups at the genus level. **Table S6.** Comparison of predicted KEGG pathways between the intestinal and non-intestinal groups. **Table S7.** The difference number of predicted functional capacities among microbiome from 12 rabbit body sites^1^.

**Additional file 2: Figure S1.** The quality plots of forward and reverse reads. The forward and reverse reads were trimmed to 281 and 206 bp, respectively. **Figure S2.** The rarefaction curves of observed ASVs and Shannon’s indexes. **Figure S3.** Evenness (pielou E) of microbial samples at 12 rabbit body sites. **Figure S4.** The comparison of alpha diversity between the intestinal group and the non-intestinal group. **Figure S5.** Comparison of rabbit microbiota at the genus level. A. Eight significantly different genera between the SSin and Lin groups. B. Three significantly different genera between the SSin and SML groups. C. Five significantly different genera between the SSin and Uter groups. D. Four significantly different genera between the Lin and SML groups. E. Three significantly different genera between the Lin and Uter groups. F. One significantly different genus between the SML and Uter groups. Welch’s t-test implemented in STAMP software was used; the *p*-values were corrected with Benjamini-Hochberg FDR method.

## Data Availability

All data generated or analyzed during this study are included in this published article [and its supplementary information files]. The 16S rRNA gene sequencing data of microbial samples from various rabbit body sites (FASTQ format) in this study have been deposited in the Genome sequence Archive in Big Data Center (http://gsa.big.ac.cn/; accession project number: PRJCA006344).

## Abbreviations

ASVs: Amplicon sequence variants
KEGG: Kyoto Encyclopedia of Genes and Genomes
KO: KEGG orthology
Lin: Large intestine
PCo: Principal coordinate
PCoA: Principal coordinate analysis
PERMANOVA: Permutational multivariate analysis of variance
SSin: Stomach and small intestine
SML: Skin, mouth and lung
UniFrac: Unique fraction metric
UPGMA: Unweighted Pair Group Method with Arithmetic mean
Uter: Uterus
