# Supplementary material for: Rabbit microbiota across the whole body revealed by 16S rRNA gene amplicon sequencing
Source: BMC Microbiol. 2021 Nov 10;21:312. doi: 10.1186/s12866-021-02377-x (PMC8579649; doi:10.1186/s12866-021-02377-x)
Supplement: Supplementary file 2 — Additional file 2: Figure S1. The quality plots of forward and reverse reads. The forward and reverse reads were trimmed to 281 and 206 bp, respectively. Figure S2. The rarefaction curves of observed ASVs and Shannon’s indexes. Figure S3. Evenness (pielou E) of microbial samples at 12 rabbit body sites. Figure S4. The comparison of alpha diversity between the intestinal group and the non-intestinal group. Figure S5. Comparison of rabbit microbiota at the genus level. A. Eight significantly different genera between the SSin and Lin groups. B. Three significantly different genera between the SSin and SML groups. C. Five significantly different genera between the SSin and Uter groups. D. Four significantly different genera between the Lin and SML groups. E. Three significantly different genera between the Lin and Uter groups. F. One significantly different genus between the SML and Uter groups. Welch’s t-test implemented in STAMP software was used; the p-values were corrected with Benjamini-Hochberg FDR method. [file 12866_2021_2377_MOESM2_ESM.docx]

**Supplementary figures:**

**
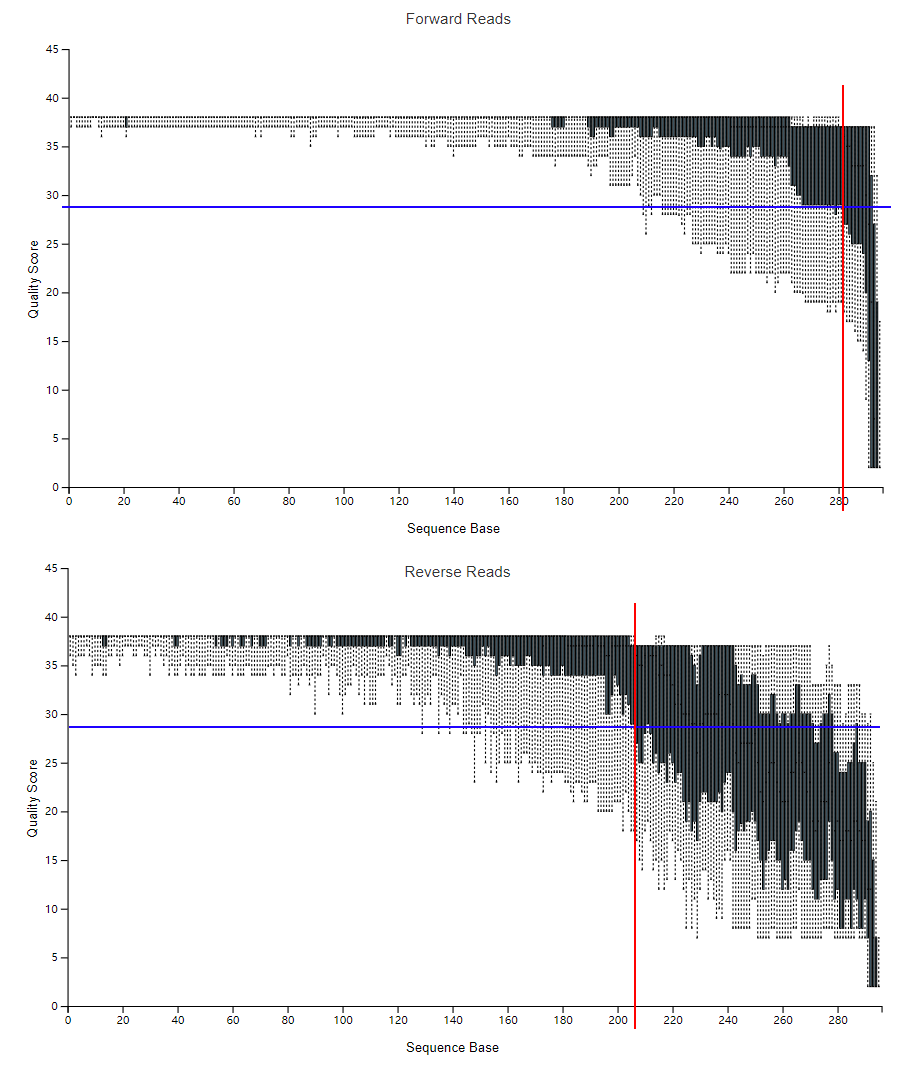
**

**Figure S1.** The quality plots of forward and reverse reads. The forward and reverse reads were trimmed to 281 and 206 bp, respectively.


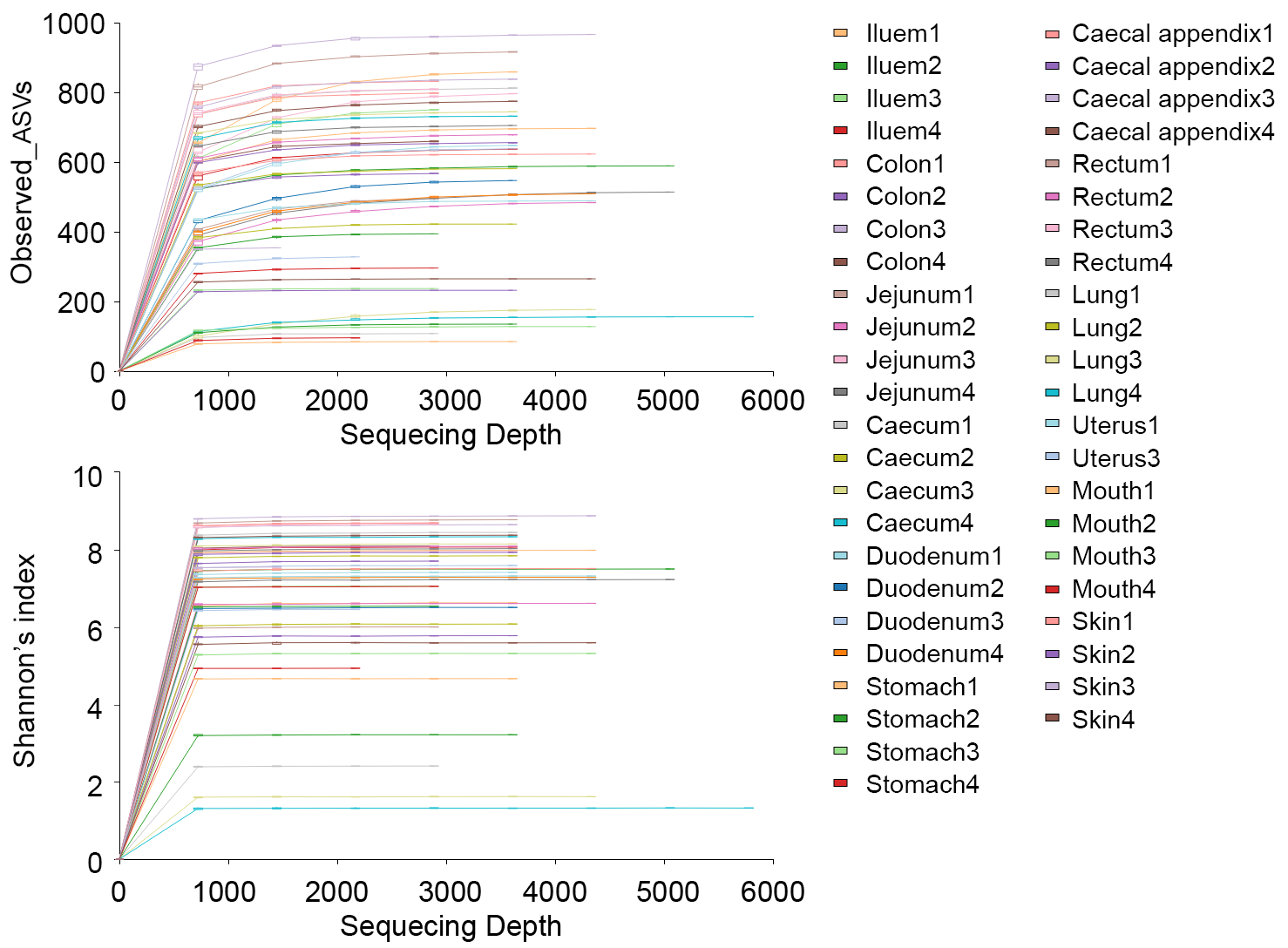


**Figure S2.** The rarefaction curves of observed ASVs and Shannon’s indexes.


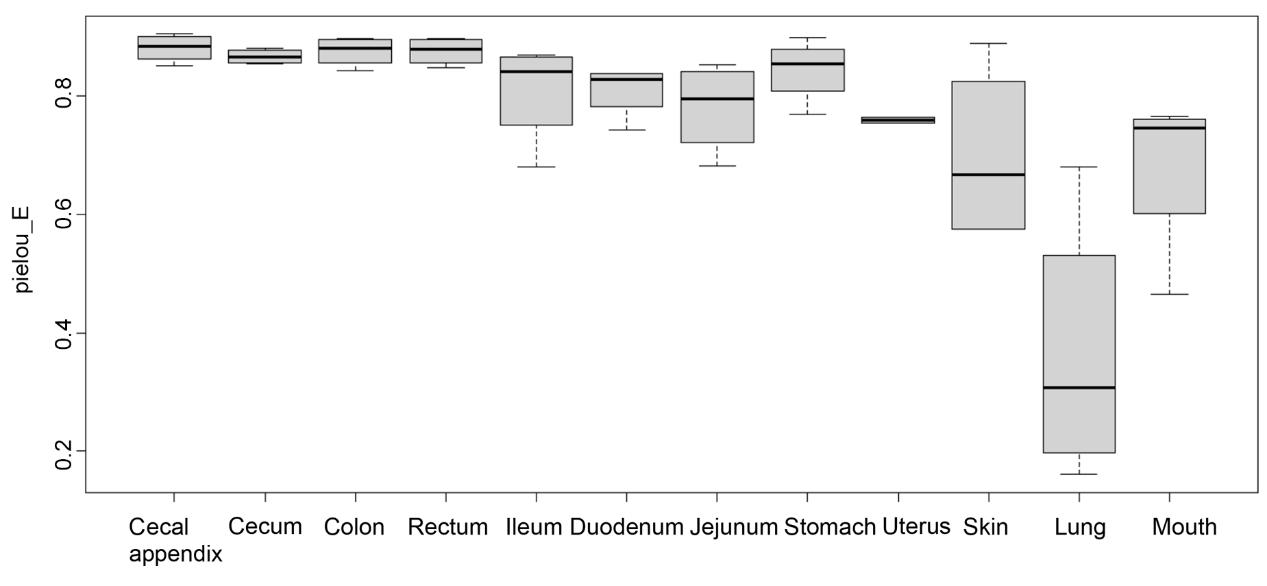


**Figure S3.** Evenness (pielou E) of microbial samples at 12 rabbit body sites.


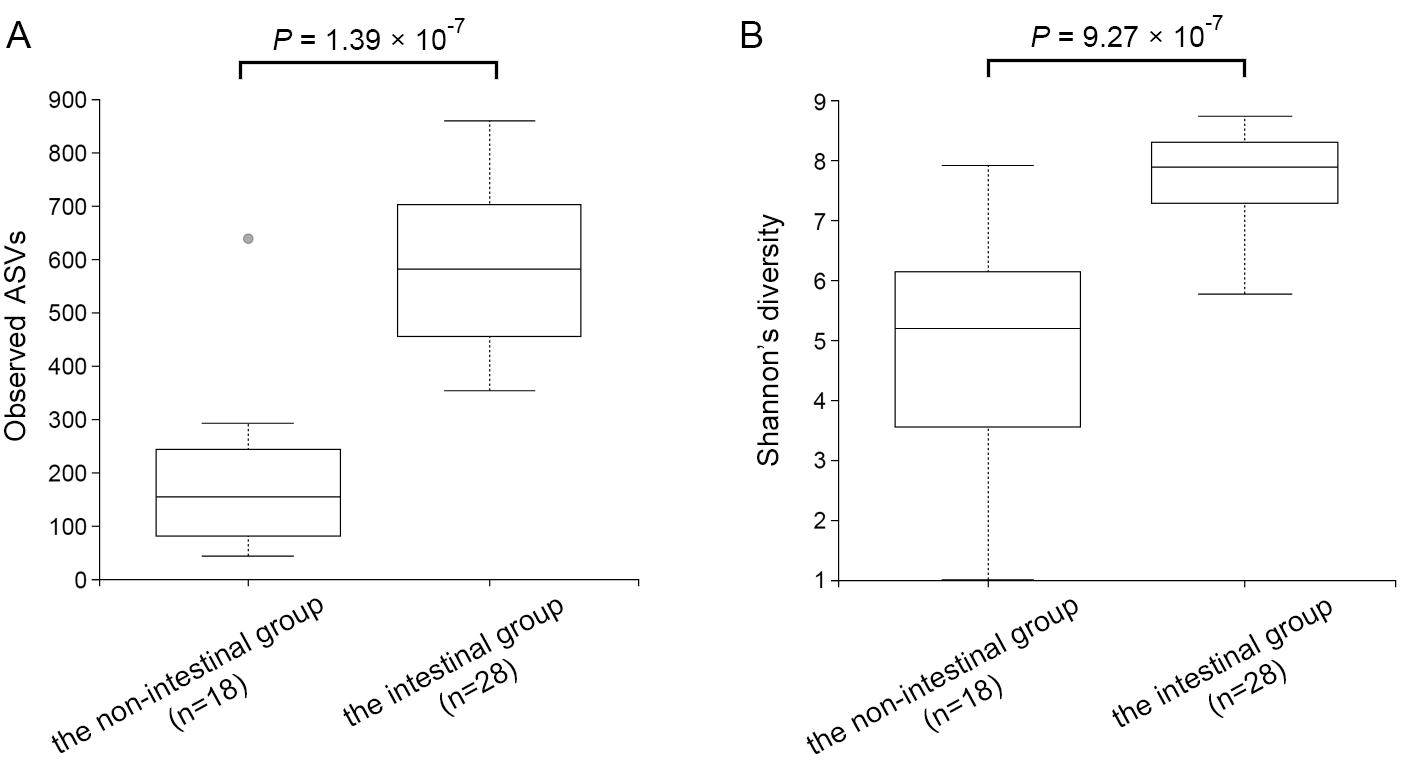


**Figure S4.** The comparison of alpha diversity between the intestinal group and the non-intestinal group.


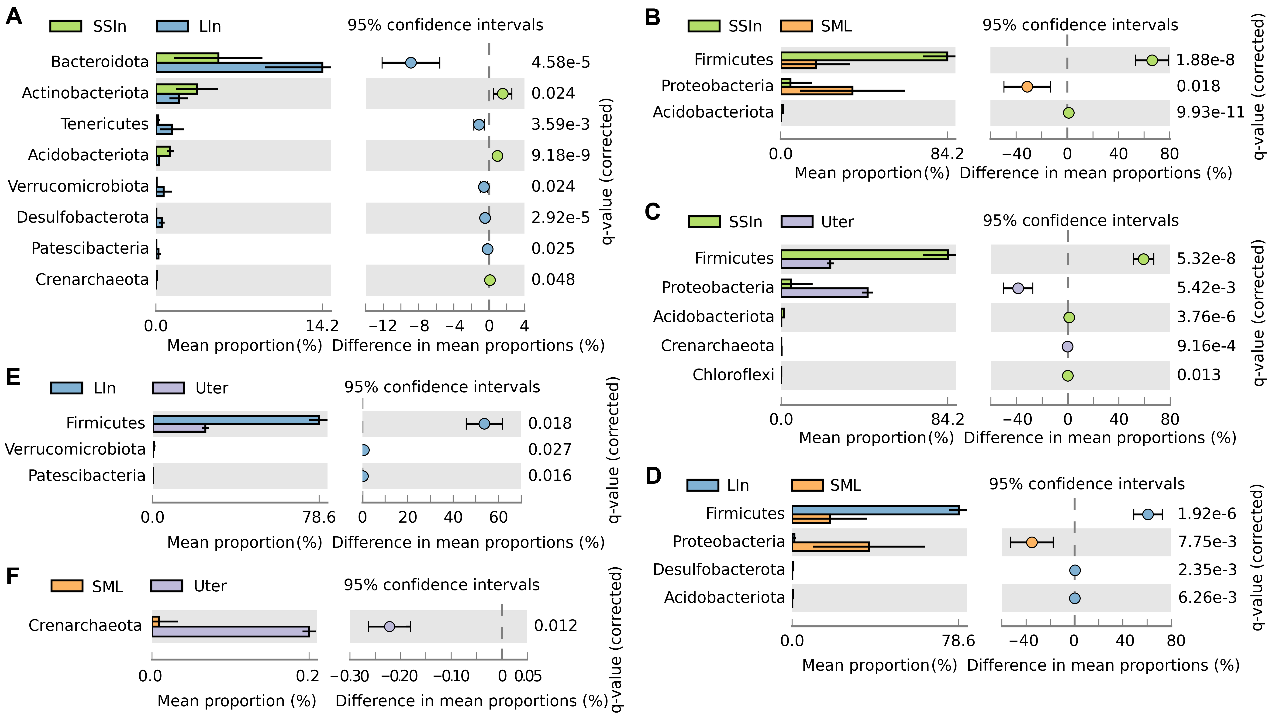


**Figure S5.** Comparison of rabbit microbiota at the genus level. A. Eight significantly different genera between the SSin and Lin groups. B. Three significantly different genera between the SSin and SML groups. C. Five significantly different genera between the SSin and Uter groups. D. Four significantly different genera between the Lin and SML groups. E. Three significantly different genera between the Lin and Uter groups. F. One significantly different genus between the SML and Uter groups. Welch’s t-test implemented in STAMP software was used; the *p*-values were corrected with Benjamini-Hochberg FDR method.
